# P55. Dendritic cell vaccination for postremission therapy in AML

**DOI:** 10.1186/2051-1426-2-S2-P29

**Published:** 2014-03-12

**Authors:** FS Lichtenegger, B Beck, I Bigalke, C Geiger, W Hiddemann, R Henschler, G Kvalheim, DJ Schendel, M Subklewe

**Affiliations:** 1 Klinikum der Universität München, Department of Internal Medicine III, Munich, Germany; 2 Helmholtz Institute Munich, Institute of Molecular Immunology, Munich, Germany; 3 Klinikum der Universität München, Division of Transfusion Medicine Cellular Therapeutics and Hemostaseology, Munich, Germany; 4 The Norwegian Radium Hospital, Department of Cellular Therapy, Oslo, Norway

## 

Cellular immunotherapy is a highly effective treatment option for patients with acute myeloid leukaemia (AML) as shown by the low relapse rate after allogeneic stem cell transplantation. However, many patients are not eligible for this treatment. This has lead to the development of various immunotherapeutic approaches that aim at inducing autologous cellular and humoral immune responses against AML and specifically against residual leukaemic stem cells (LSCs).

Dendritic cells (DCs) are important regulators of the human immune response. We have developed a three-day DC manufacturing protocol that starts with peripheral blood monocytes, e.g. from AML patients in remission following intensive chemotherapy. By using a cytokine cocktail containing a synthetic TLR7/8 agonist, the resulting DCs develop improved immunogenicity. For healthy donors as well as for AML patients, we were able to show that these DCs display a positive costimulatory profile, secrete high levels of IL-12p70, show chemotaxis to CCR7 ligands, and activate NK cells. After loading the DCs with mRNA, they effectively induce antigen-specific T cell responses with a strong type-1 polarization. Due to these properties, this DC type seems highly suitable for application in cancer immunotherapy.

We have recently initiated a phase I/II clinical trial for the application of these DCs in the setting of AML postremission strategy. WT1 and PRAME were chosen as leukaemia-associated antigens due to their overexpression on leukaemic blasts and specifically on cells that are enriched for LSCs. DCs transfected with mRNA encoding CMV-pp65 are included into the vaccine as an adjuvant as well as a surrogate antigen. 20 patients with a non-favourable risk profile or with confirmed minimal residual disease (MRD), but who are not eligible for allogeneic stem cell transplantation, will be included. The primary objective of this study is to evaluate feasibility and safety of this immunotherapeutic approach. Important secondary endpoints are immune responses to the applied antigens and MRD control. First results of this study will be presented.

